# Resmetirom: The First Disease-Specific Treatment for MASH

**DOI:** 10.1155/ije/6430023

**Published:** 2025-02-26

**Authors:** Ali Mohamed Mousa, Mervat Mahmoud, Ghaida Mubarak AlShuraiaan

**Affiliations:** Family Medicine Department, Ahmadi Hospital, Kuwait Petroleum Corporation, Al Ahmadi, Kuwait

## Abstract

In 2023, the medical terminology for Nonalcoholic Fatty Liver Disease (NAFLD) and NA SteatoHepatitis (NASH) was updated to Metabolic Dysfunction-Associated SteatoticLiver Disease (MASLD) and MA SteatoHepatitis (MASH). This review highlights the critical epidemiological, pathophysiological, and therapeutic aspects of MASH, focusing on the novel treatment option, resmetirom. Resmetirom, a thyroid hormone receptor-beta (THR-β) agonist, specifically targets liver function to simulate localized hyperthyroidism, effectively reducing lipid accumulation and liver fibrosis without the systemic effects commonly associated with thyroid hormone therapy. Clinical trials, particularly the pivotal MAESTRO-NASH trial, have demonstrated significant improvements in liver health metrics, achieving primary endpoints by resolving MASH without worsening fibrosis and showing a favorable safety profile. This paper reviews the mechanism of action, efficacy, and safety of resmetirom, providing insight into its potential to change the therapeutic landscape for patients suffering from MASH.

## 1. Introduction

In 2023 [1], the medical community updated the terms Nonalcoholic Fatty Liver Disease (NAFLD) and NA SteatoHepatitis (NASH) to Metabolic Dysfunction-Associated Steatotic Liver Disease (MASLD) and MA SteatoHepatitis (MASH). Endorsed by liver and metabolic disease experts from 56 countries, this renaming highlights the role of metabolic dysfunction in these conditions and aims to reduce stigma [2].

MASLD is diagnosed with fat accumulation in the liver accompanied by metabolic disturbances such as obesity, diabetes, high blood pressure, elevated triglycerides, or low HDL cholesterol. MASH, a more severe form, involves additional liver inflammation and damage, potentially leading to complications like fibrosis, cirrhosis or hepatocellular carcinoma [3].

Affecting over 30% of the global population, MASLD presents significant health and economic challenges, particularly in the United States, where costs exceed $100 billion annually. Its prevalence is notably higher in regions like the Middle East and North Africa, significantly influenced by type 2 diabetes primarily due to insulin resistance. Approximately 65.26% of patients with type 2 diabetes also suffer from MASH, reflecting a significant overlap in pathophysiological mechanisms [4].

This review aims to provide a comprehensive overview of emerging therapeutic strategies for MASH, focusing on treatments that target its multifactorial pathogenesis. We discuss the mechanisms of action, efficacy, and safety of novel therapies, with particular emphasis on resmetirom, the first FDA-approved treatment for MASH.

## 2. Pathophysiological Insights

MASH is characterised by hepatic steatosis, inflammation, and cellular damage. The pathogenesis involves insulin resistance leading to lipid accumulation in liver cells, followed by lipotoxicity, which induces cellular stress and contributes to liver cell damage. Chronic immune system activation releases pro-inflammatory cytokines, exacerbating liver injury. Persistent inflammation activates fibrogenesis, where ongoing damage causes scar tissue formation, potentially leading to cirrhosis. Genetic factors, notably the PNPLA3 polymorphism, significantly influence the susceptibility and progression of MASH [5, 6].

Emerging evidence highlights the role of ATP Citrate Lyase (ACLY) in the progression of MASH. Overexpression of ACLY in hepatocytes contributes to lipid accumulation and oxidative stress, exacerbating liver inflammation and fibrosis. Inhibition of ACLY has been shown to reverse lipid peroxidation and reduce inflammatory cytokines like IL-6 and IL-1β, suggesting its potential as a therapeutic target in MASH management [7].

Pro-inflammatory cytokines, particularly TNF-α, play a pivotal role in the progression of MASH. Elevated levels of TNF-α in patients with MASH contribute to insulin resistance and fibrogenesis, perpetuating the inflammatory cycle and promoting liver fibrosis. These findings reinforce the link between chronic inflammation and liver damage in MASH [8].

This development from initial metabolic dysfunction to severe liver disease follows the “multiple hit” theory. This theory posits that multiple factors—including insulin resistance, adipose tissue dysfunction, chronic inflammation, oxidative stress, and changes in gut microbiota—occur in sequence or simultaneously in genetically predisposed individuals, exacerbating the disease progression. The presence and severity of fibrosis, resulting from chronic hepatic inflammation and the body's healing response, are critical in determining the progression to cirrhosis, with steatohepatitis serving as a principal driver of fibrosis development [9].

## 3. Treatment Approaches

Management primarily focuses on lifestyle interventions, such as dietary modifications and increased physical activity, which remain the cornerstones of MASH treatment. Weight loss of 5%–10% has been shown to significantly reduce hepatic steatosis and improve fibrosis markers, making it a critical component of therapy [10]. However, sustained weight loss is challenging, and for patients where lifestyle changes are insufficient, pharmacological treatments and bariatric surgery are considered [10, 11].

Pharmacological interventions have gained increasing importance, especially with the FDA approval of resmetirom, a thyroid hormone receptor-beta (THR-β) agonist, as the first disease-specific treatment for MASH [12]. Beyond resmetirom, other promising drug classes are being investigated, including GLP-1 receptor agonists (GLP-1RAs), peroxisome proliferator-activated receptor (PPAR) agonists, fibroblast growth factor (FGF) analogues, and sodium-glucose cotransporter-2 (SGLT2) inhibitors, all of which target various pathophysiological pathways of MASH [13].

### 3.1. GLP-1 Receptor Agonists (GLP-1RAs)

GLP-1RAs have shown considerable promise in MASLD and MASH management, particularly by addressing key metabolic dysfunctions such as obesity, insulin resistance, and hepatic steatosis. In a randomized clinical trial, liraglutide resulted in a 39% resolution of NASH without worsening fibrosis, compared to 9% in the placebo group [14]. Similarly, semaglutide demonstrated NASH resolution rates of 59% in patients treated with 0.4 mg daily, compared to 17% in the placebo group, without significant worsening of fibrosis [15]. These findings underscore the anti-inflammatory and hepatoprotective effects of GLP-1RAs, making them strong candidates for future MASH therapy.

### 3.2. Dual and Triple Agonists: Expanding the Therapeutic Landscape

Beyond GLP-1 receptor agonists, novel dual and triple agonists are being developed to target multiple metabolic pathways simultaneously, providing superior benefits in hepatic steatosis reduction and metabolic control [13].

Tirzepatide (GLP-1/GIP dual receptor agonist): In a 26-week study involving patients with type 2 diabetes, tirzepatide significantly reduced NASH biomarkers, including ALT by 16.4 U/L and AST by 7.2 U/L at the highest 15 mg dose (*p* < 0.05). Additionally, hepatic fat fraction decreased by up to 16%, demonstrating its efficacy in reducing liver steatosis [16].

Retatrutide (GLP-1/GIP/glucagon receptor agonist): A Phase two trial showed that patients treated with 12 mg retatrutide experienced a 24.2% reduction in body weight after 48 weeks, which translated into significant liver fat reduction. MRI-PDFF measurements confirmed a 38% decrease in hepatic fat content, reinforcing the potential benefits of polyagonists for metabolic and liver health [17].

### 3.3. PPAR Agonists: Targeting Lipid Metabolism and Inflammation

PPAR agonists play a key role in MASH treatment, modulating lipid metabolism, insulin sensitivity, and fibrosis progression [13].

Lanifibranor (pan-PPAR agonist): The NATIVE trial demonstrated that lanifibranor significantly improved fibrosis and metabolic parameters, making it a viable therapeutic candidate for MASH [18].

Saroglitazar (PPAR-γ/*α* dual agonist): Shown to reduce ALT levels, hepatic fat content, and insulin resistance, saroglitazar is currently being evaluated as a potential treatment for MASH patients with metabolic comorbidities [19].

### 3.4. FGF Analogues

FGF analogues are an emerging class of therapies that modulate hepatic lipid metabolism, glucose homeostasis, and fibrosis progression [13].

Efruxifermin (FGF-21 analogue): A Phase two trial reported significant reductions in liver fat and fibrosis improvement, supporting its potential role in MASH management [20].

Pegbelfermin (FGF-21 analogue): In the FALCON 1 trial, pegbelfermin reduced hepatic fat fraction (MRI-PDFF), improved fibrosis markers (ELF score, FIB-4, APRI), and lowered fibrogenesis biomarkers (PRO-C3, CK-18), suggesting benefits in liver steatosis and fibrosis in NASH patients with stage 3 fibrosis [21].

### 3.5. SGLT2 Inhibitors: A New Avenue in MASH Therapy

SGLT2 inhibitors, primarily used for type 2 diabetes, have shown potential in MASLD and MASH by reducing hepatic steatosis and improving insulin sensitivity [13]. Moreover, in a recent study, SGLT2 inhibitors have been shown to exert protective effects against liver fibrosis by modulating key metabolic pathways. These findings suggest that SGLT2 inhibitors may offer therapeutic benefits in managing MASH by addressing both metabolic dysfunction and fibrotic progression [22].

Once considered promising, other agents like statins, omega-3 fatty acids, and silymarin have not shown consistent benefits in improving liver outcomes in MASH. They are not generally recommended for liver disease alone [23–25].

### 3.6. Thyroid-Liver Interactions and Implications

**Figure 1:**
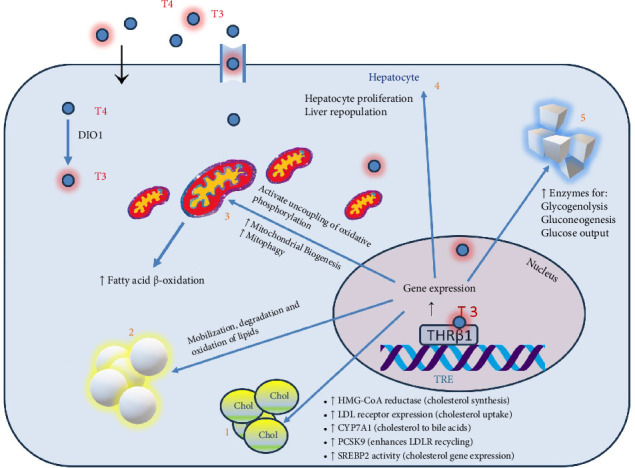
The genomic effect of thyroid hormone on hepatocytes [26–30]. T3 and T4 enter the hepatocyte through simple diffusion and cell membrane transporters. T4 is converted to T3 by deiodinase 1 (top left). THR-β is a member of the nuclear hormone receptor family, which binds to the thyroid response element (TRE) in the DNA. T3 binding to THR-β1 leads to transcription of several key genes, which affects (1) cholesterol metabolism, (2) lipid metabolism, (3) mitochondrial biogenesis and function, (4) hepatocyte proliferation and (5) carbohydrate metabolism. Abbreviations: Chole, cholesterol; DIO1, deiodinase 1; T3, tri-iodothyronine; T4, thyroxine; THR-β1, thyroid hormone receptor beta 1; TRE, thyroid response element.

The liver and thyroid gland are fundamentally interlinked, with each organ influencing the function of the other through complex metabolic interactions (Figure 1). These interactions are evident across various thyroid functional states, significantly impacting liver health and disease dynamics.

In states of hypothyroidism, the liver's lipid metabolism is often disrupted, resulting in elevated levels of total cholesterol, LDL cholesterol, and triglycerides. Moreover, thyroid hormones play a critical role in liver regeneration; a deficiency can impair the liver's ability to repair itself post-injury or surgery. There is also evidence that hypothyroidism may elevate the risk of liver fibrosis in MASLD patients, possibly due to prolonged oxidative stress and lipid peroxidation [31].

Conversely, hyperthyroidismincreases the overall metabolic rate, which can elevate liver enzyme levels, occasionally misinterpreted as liver damage. While severe hyperthyroidism can cause hepatic dysfunction, including hepatomegaly and elevated liver enzymes, these conditions typically resolve with effective thyroid disorder management. Hyperthyroidism might offer protective effects against liver fat accumulation due to increased basal metabolic rate and enhanced lipid utilization [32].

An important common feature is impaired conversion of thyroxine (T4) to triiodothyronine (T3) in the hepatic tissue in people with MASLD, which resembles a “localized hypothyroidism” in the liver [33].

### 3.7. THR and Their Role in Liver Disease Treatment

THRs, including THR alpha (THRα) and THR beta (THRβ), are nuclear receptors that control the actions of thyroid hormones like T3 [34]. Given the hallmark lipid disturbances in MASLD, activating THRβ receptors could help normalize these metabolic disruptions. Selective stimulation of THRβ receptors in the liver, avoiding non-selective thyroid hormone stimulation effects, has been proposed as a therapeutic strategy to improve liver lipid profiles without systemic side effects [35].

## 4. Resmetirom

Resmetirom functions as a THR-β agonist, specifically targeting liver function to achieve what can be described as “localized hyperthyroidism” within the liver. This targeted action stimulates lipid metabolism and reduces lipid accumulation and liver fibrosis, which are critical aspects of NASH management [36].

The pivotal MAESTRO-NASH trial [37], a Phase 3, randomized, double-blind, placebo-controlled study, assessed the efficacy of Resmetirom in patients with biopsy-confirmed NASH and fibrosis stages F2 to F3. The study involved 1444 participants who received either 80 mg, 100 mg of Resmetirom, or a placebo daily over 52 weeks. Patients were evaluated using a combination of liver biopsies, MRI-Proton Density Fat Fraction (MRI-PDFF), and blood biomarker analyses to provide a comprehensive assessment of the treatment's effects. Liver biopsies were performed at baseline and at the 52-week mark to assess histological changes, with primary endpoints including resolution of NASH without worsening fibrosis and improvement in fibrosis by at least one stage without worsening of NASH. The results showed that 24.2% of patients in the 80 mg group and 25.9% in the 100 mg group achieved at least one-stage fibrosis improvement without worsening NASH, compared to 14.2% in the placebo group (*p* < 0.001). NASH resolution was determined using the NAFLD Activity Score (NAS), which evaluates steatosis, inflammation, and hepatocellular ballooning; resolution was defined as reducing ballooning to zero and inflammation to zero or one, indicating no active NASH. MRI-PDFF was employed as a non-invasive imaging method to quantify hepatic fat content, revealing that Resmetirom led to a 26.7% reduction in liver fat at the 80 mg dose and a 37.9% reduction at the 100 mg dose after 52 weeks. Blood biomarkers, including liver enzymes (ALT, AST, GGT) and lipid profile markers (LDL cholesterol, triglycerides), were also monitored throughout the study, with improvements correlating with the imaging and histological findings, providing additional evidence of Resmetirom's impact on liver function and metabolic health. This comprehensive approach confirmed Resmetirom's efficacy in treating NASH and liver fibrosis, with improvements observed in both histological and functional liver health, reinforcing the drug's potential clinical benefits for NASH patients.

The MAESTRO-NASH trial also highlighted Resmetirom's potential cardiovascular benefits. The trial showed reductions in LDL cholesterol by 13.6% for the 80 mg dose and 16.3% for the 100 mg dose, compared to minimal changes in the placebo group. Given that cardiovascular disease is a major comorbidity in MASH, these findings underscore Resmetirom's potential to address both liver-specific and broader metabolic risks associated with MASH [12].

Other than the MAESTRO-NASH trial, the ongoing MAESTRO clinical program [38] is a comprehensive Phase 3 initiative designed to thoroughly evaluate Resmetirom for the treatment of nonalcoholic steatohepatitis (NASH) by exploring both histological and clinical outcomes essential for regulatory consideration. This program includes several trials that assess Resmetirom's impact on liver health through a combination of invasive and non-invasive methods, reflecting a detailed approach to evaluating its potential as a targeted therapy. Trials such as MAESTRO-NAFLD-1 and MAESTRO-NAFLD-OLE utilize MRI-Proton Density Fat Fraction (MRI-PDFF) to monitor reductions in hepatic fat content, emphasizing non-invasive diagnostic techniques to support broader clinical applicability. At the same time, MAESTRO-NASH-OUTCOMES extends this evaluation to patients with well-compensated NASH cirrhosis to understand the drug's role in preventing progression to severe complications. This extensive trial design aligns with regulatory expectations for accelerated approval by focusing on meaningful histological endpoints while also gathering data on secondary outcomes such as lipid changes and cardiovascular health. The preliminary data have been promising, and as the program continues, it aims to deliver comprehensive insights into Resmetirom's effectiveness and its potential to offer a new therapeutic pathway for NASH management.

Resmetirom has shown a generally favorable safety profile, with most adverse events reported as mild to moderate. The most common side effects observed in clinical trials were gastrointestinal, such as diarrhoea and nausea, which occurred at higher rates in patients receiving Resmetirom compared to those on a placebo. These symptoms were generally transient and manageable without discontinuation of the drug [39].

A notable aspect of Resmetirom's safety profile is its selective action on THR-β, which primarily affects liver metabolism with minimal impact on systemic thyroid hormone levels. While Resmetirom has been associated with reductions in free T4 (FT4) by around 16%–19%, it does not significantly affect free T3 (FT3) or thyrotropin levels, reducing the risk of systemic hypothyroidism [40]. This selective mechanism of Resmetirom reduces the likelihood of adverse effects on other tissues, such as the heart and bones, which are predominantly regulated by THR-alpha (THR-α). Since THR-β is primarily active in the liver, Resmetirom's targeted action ensures that systemic thyroid hormone levels remain largely unaffected, thereby minimizing risks typically associated with THR-α activation in cardiovascular and skeletal tissues [41].

Despite its favorable safety profile, there have been rare cases of hepatotoxicity linked to Resmetirom. Although severe liver injury has not been observed, some patients have experienced asymptomatic increases in liver enzymes. Given this, routine monitoring of liver function is recommended, particularly in patients with pre-existing liver conditions or those on concurrent medications with potential hepatotoxic effects [12, 42].

Resmetirom also has potential drug interactions, particularly with medications metabolized by liver enzymes, as it is processed through the cytochrome P450 (CYP) pathway. Drugs that are strong inducers or inhibitors of CYP enzymes—such as CYP3A4, CYP2C8, and CYP2C9—could potentially affect Resmetirom's metabolism and efficacy. For example, statins, commonly prescribed to manage cholesterol in patients with MASH, are metabolized by these enzymes. When Resmetirom is used concurrently with statins, there is an increased potential for adverse effects like muscle toxicity or hepatotoxicity. To mitigate these risks, dose adjustments of statins may be necessary. Clinicians might consider reducing the statin dose or monitoring liver enzymes and muscle symptoms more closely when both medications are concurrently prescribed [43].

The American Diabetes Association (ADA), in its latest guidelines [44], highlights Resmetirom as a groundbreaking treatment for MASH, particularly in individuals with type 2 diabetes or prediabetes and moderate to advanced liver fibrosis. The ADA highlights its dual benefits in improving liver health and systemic metabolic risks, including reductions in hepatic fat, fibrosis progression, and LDL cholesterol levels, which may help mitigate cardiovascular risks. While recognizing its favorable safety profile, the ADA underscores the need for routine liver function monitoring due to rare cases of asymptomatic liver enzyme elevations. Additionally, the ADA recommends that treatment initiation and management be overseen by hepatologists or gastroenterologists, ensuring careful patient selection and monitoring.

## 5. FDA Approval and Clinical Implications

The FDA granted accelerated approval to resmetirom as the first medication to treat NASH with liver fibrosis, addressing a significant unmet medical need. This approval was based on comprehensive data from 18 clinical studies, including Phase 1, Phase 2, and Phase 3 trials that demonstrated the drug's effectiveness in improving liver health [45].

The FDA approval of Resmetirom has profound implications across multiple domains. As the first approved treatment for NASH, Resmetirom offers a targeted approach previously unavailable, addressing a significant gap in managing this liver disease associated with metabolic dysfunction [46, 47]. This approval marks a paradigm shift, as NASH can now be managed with a liver-specific therapy alongside lifestyle modifications. By reducing hepatic fat content, Resmetirom aims to resolve NASH and mitigate fibrosis, which is expected to significantly improve patient outcomes [48]. For those with advanced fibrosis, the drug may reduce the need for liver transplants and other invasive interventions. Furthermore, Resmetirom's efficacy in lowering LDL cholesterol levels provides additional benefits by potentially reducing cardiovascular risks, a significant concern given the high prevalence of cardiovascular comorbidity among NASH patients. This dual action enhances Resmetirom's value as a comprehensive therapeutic option [49].

The approval also supports the adoption of noninvasive diagnostic methods, reducing the need for liver biopsies. This could expedite the shift toward noninvasive diagnostics, improving patient access and safety. Regarding economic and public health impact, Resmetirom may help lower healthcare costs associated with NASH management; however, considerations of the drug's pricing and reimbursement policies will be critical for its accessibility, particularly in underserved populations [50]. Additionally, the success of Resmetirom has highlighted THR-β agonists as a promising class of drugs, which could inspire further research into thyroid hormone pathways for treating other metabolic conditions beyond NASH, such as dyslipidemia and obesity [51].

## 6. Conclusion

Resmetirom's FDA approval is transformative for both NASH patients and the broader field of hepatology and metabolic medicine. Its potential benefits in improving liver health and reducing cardiovascular risk by directly targeting the underlying mechanisms of the disease position it as a valuable therapeutic option. The approval's implications extend into healthcare policy, diagnostic practices, and ongoing research, underscoring Resmetirom's role in managing NASH and potentially influencing the future landscape of metabolic disease treatment.

## Data Availability

Data sharing is not applicable to this article as no new data were created or analyzed in this study.
